# ELISA Test Based on the Phenolic Glycolipid-I (PGL-I) of *Mycobacterium leprae*: A Reality of a Laboratory from a Non-Endemic Country

**DOI:** 10.3390/pathogens11080894

**Published:** 2022-08-09

**Authors:** Silvia Stefania Longoni, Anna Beltrame, Marco Prato, John Stewart Spencer, Nicolo Bergamaschi, Andrea Clapasson, Aurora Parodi, Chiara Piubelli, Francesca Perandin

**Affiliations:** 1 Department of Infectious, Tropical Diseases and Microbiology, IRCCS Sacro Cuore Don Calabria Hospital, 37024 Verona, Italy; 2 Mycobacteria Research Laboratories, Department of Microbiology, Immunology and Pathology, Colorado State University, Fort Collins, CO 80523, USA; 3 Dermatological Clinic, National Reference Center for Hansen’s Disease, IRCCS Ospedale Policlinico San Martino, 16132 Genoa, Italy; 4 Department of Health Science (DISSAL), University of Genova, 16132 Genoa, Italy

**Keywords:** ND-O-BSA, leprosy, ELISA, Europe

## Abstract

Background: Leprosy is a neglected tropical disease caused by *Mycobacterium leprae*, leading to disabilities if untreated. The ELISA based on phenolic glycolipid I (PGL-I), or its synthetic version ND-O-BSA, is almost universally positive in multibacillary leprosy and thus extensively used in endemic countries. Household contacts with a positive antibody titer have ~6-fold higher probability to develop the disease than those with a negative titer. Thus, the aim of the study was to evaluate the performance of this ELISA in the setting of a non-endemic country. Methods: We calculate the cut-off using optimized O.D. thresholds, generated by receiver operating characteristics (ROC) curve analysis, testing 39 well-characterized sera obtained from lepromatous leprosy patients with strongly positive ND-O-BSAELISA titer and 39 sera from healthy non-endemic patients never exposed to *M. leprae* or *M. tuberculosis*. Indeed, we tested a second set of sera from suspected or confirmed leprosy or household contacts (SLALT group, n=50), and patients with tuberculosis (control group, n=40). Results: We detected 56.4% of SLALT and 22.5% of tuberculosis as positive, consistent with the literature. Conclusion: The ELISA based on ND-O-BSA may thus be considered a good option to be used in a non-endemic area as a screening tool in at risk population usually coming to our center.

## 1. Introduction

Leprosy, or Hansen’s Disease (HD), is a neglected tropical disease (NTD) caused by *Mycobacterium leprae*. If untreated, chronic *M. leprae* infection can result in skin lesions and progressive and permanent nerve damage, deformity and disability with resulting social stigma. According to the World Health Organization (WHO), leprosy is endemic in 22 countries with a worldwide incidence of more than 200,000 new cases per year for over 10 years [1,2,3] with 80% of the total number of new cases detected each year found in just three countries: Brazil, Indonesia and India. In Europe, the WHO records on average 20 cases per year, but data for all EU countries are not available [1]. In Italy, leprosy is a rare infectious disease, almost exclusively found in individuals coming from endemic countries [4]. 

*M. leprae* infection has a long incubation period before becoming manifest and only around 10% of infected individuals will eventually develop recognizable clinical symptoms, most within a 2-6-year timeframe. Nevertheless, during asymptomatic or subclinical infection, patients can transmit the bacteria [5]. To reduce the risk of *M. leprae* dissemination, it is necessary to perform confirmatory laboratory tests that can detect early biomarkers of infection. Early detection of new cases remains the fundamental principle for leprosy control [6].

Currently, the diagnosis of leprosy is mainly based on the clinical observation of cutaneous and neuritic signs [7,8]. However, the modality of clinical diagnosis are not easy to perform by non-specialised clinicians [9]. Leprosy is classified following the number of skin lesions: paucibacillary (PB) if presenting five or less skin lesions or multibacillary (MB) if ˃5 lesions. The diagnosis could be confirmed by histological examination of affected skin or nerve [7]. Using Ridley–Jopling histological classification allows for a more accurate diagnosis, although the results deeply depend on the site from which the biopsy has been taken [10,11]. However, microscopic observations of bacilli after Ziehl–Nielsen coloration in the skin and nerves biopsies, slit-skin smears (SSS) or nasal swab have a very low sensitivity (30–40%) [8,12,13]. Polymerase chain reaction (PCR) can reach higher sensitivity than microscopy, resulting in a confirmatory test [14]. Unfortunately, this tool is not always available in resource limited laboratories and the maximum sensitivity is achieved when the disease has already progressed to an advanced stage. 

Serological tests represent an available, valid, and cheaper tool that could overcome the sensitivity problems. The aim of many studies has been to identify a specific and highly sensitive antigen as a biomarker for a serodiagnostic test for leprosy. Because *M. leprae* and *Mycobacterium tuberculosis* present a wide number of homologies, sharing 90% of their coding-genes [15], most of the suggested antigenic fractions presented cross-reactions as reflections of these homologies. Amongst the hundreds of possible *M. leprae* antigenic fractions that have been tested so far [16], only a few have shown high specificity for *M. leprae*. The native phenolic glycolipid I (PGL-I), or its synthetic version, ND-O-BSA, is one of these [17]. It is recognized by human IgM antibodies and it has already been included in an enzyme-linked immunosorbent assay (ELISA) which shows limited diagnostic and prognostic capabilities if a single determination is made. Household contacts (HC) of leprosy patients who have positive PGL-I serology have approximately six-fold higher probability to develop the disease in the following five years than those with negative serology [18,19]. The ELISA based on PGL-I has been extensively used in endemic countries [20,21], but is not commercially available. 

The present study aims to demonstrate the utility to implement the in-house ELISA based on PGL-I as screening program in those patients at risk to develop leprosy due to their travel history, and indeed to obtain a more accurate follow up consistent with the next years in order to prevent any worst scenario of physical damage and consequently social discrimination.

## 2. Materials and Methods

### 2.1. Subjects and Samples

A total of 168 serum samples, divided into two different sets, were included in the study. The summary of the subjects’ characteristics is reported in Table 1.

Table 1. Summary of the subjects’ characteristics. Legend: Positive and Negative: Respectively: Positive and Negative Control Group; SLALT: Suspected Leprosy Or After Leprosy Treatment Cases; TB: Patient with laboratory confirmed tuberculosis; WHO classification: PB: Paucibacillary, ≤5 skin lesions, SSS all negative and MB: Multibacillary, >5 skin lesions, SSS positive. Ridley–Jopling classification: BL: borderline lepromatous leprosy; LL: Lepromatous leprosy; BB: mid-borderline leprosy; TT, polar tuberculoid; BT, borderline tuberculoid; I, indeterminate; PNL, pure neural leprosy, LL-R: Lepromatous leprosy reaction. QFT-Plus: Quantiferon-Plus Test; N/A: Not Available; Neg: Negative; Pos: Positive. Sex M/F = Masculine/Feminine; IQR: interquartile range.* Positive = positive results also to PGL-I ELISA performed as published in Spencer et al. 2011 [22]. 

#### 2.1.1. First Set

- Positive control group: 39 sera of lepromatous leprosy patients (11 BL and 28 LL forms) from an endemic country (Philippines rural areas) [17]. Diagnosis was based on well-accepted clinical signs and symptoms performed by experienced leprologists and a leprosy pathologist.
- Negative control group: 39 sera of healthy controls from a non-endemic country (Italy). These were healthy volunteers with no *M. tuberculosis* disease or latent infection as all tested negative for the QuantiFERON-TB Gold Plus test (QIAGEN, Hilden, Germany) and were also without any exposure to a leprosy patient.

#### 2.1.2. Second Set

- SLALT (Suspected Leprosy or After Leprosy Treatment) group: 50 sera from leprosy patients (travellers/migrants), under treatment or who had completed at least one course of treatment, cured in a nonendemic country (Italy) and patients with possible signs and symptoms suspected of leprosy as well as household contacts of diagnosed cases. Diagnosis was based on laboratory (microscopic observation of acid-fast bacilli (AFB) from skin lesions or PCR positive) and clinical data performed by experienced leprologists.
- TB (Tuberculosis) group: 40 sera from patientsresident in Italy with active disease caused by *M. tuberculosis* (tuberculosis) confirmed by real-time qPCR [23], before the start of the TB treatment.

### 2.2. Enzyme-Linked Immunosorbent Assay (ELISA)

In this study we use the protocol currently used in endemic countries and previously published by Spencer and co-workers [22], with slightly modifications. Briefly, ND-O-BSA antigen was coated onto high-affinity polystyrene Immulon IV 96-well ELISA plates (ThermoFisher, Waltham, MA, USA) using 25 ng/well in 100 µl of 0.1M sodium carbonate/bicarbonate buffer, pH 9.6, at 4 °C overnight. Unbound antigen was washed away and the wells were blocked using a blocking buffer containing PBS (pH 7.4), 1% BSA and 0.05% Tween 80 and incubated for 1½ hours at room temperature. Serum samples were diluted 1:300 in blocking buffer and 100 µl were added to the wells, each serum sample was tested in at least two replicates, and incubated for 2h at room temperature. After incubation with the primary antibody, the wells were washed with PBS with 0.05% Tween 80 (wash buffer), followed by the addition of 100µl of a 1:10.000 dilution of the secondary anti-human polyvalent antibody (Sigma-Aldrich A-3313, Merck KGaA, Darmstadt, Germany) for 30min at 37 °C. After washing the wells with PBS six times, 50 µl of Alkaline Phosphatase Substrate (Sigma-Aldrich P-7998, Merck KGaA, Darmstadt, Germany) was added. The absorbance at 405 nm was read using a ELx800 plate reader (Biotek, Winooski, VT, USA) after 15 min of dark incubation at 37 °C.

We tested serially diluted antigen concentrations from 1 ng/well to 0.48 ng/well using a 1/300 pooled positive control serum dilution. We also tested different sera dilutions, from 1/100 to 1/3200, using 25ng/well ND-O-BSA concentration and two different concentrations of secondary antibody: 1/10,000 and 1/20,000 using 25ng/well of ND-O-BSA, and 1/300 diluted positive control pool. The best performing concentration of antigen and serum dilution was then applied to the in-house ELISA.

After testing the second set of serum samples, we applied the corresponding cut-off value, and we normalized the score based on the equation “O.D. Samples/cut-off”. If the results were ≥1 it was considered as positive, whereas if <1 it was considered negative (Supplementary 1). In this particular scenario we did not consider a possible grey zone (0.9 < x < 1) which should be addressed in future studies.

### 2.3. Statistical Analysis

All collected data were summarized using descriptive statistics. Estimated parameters are reported with 95% confidence interval (CI). The statistical significance level was established at 5%. Both statistical methods and plots were used to assess test results. Optical density (O.D.) values of the replicates were evaluated using the coefficient of variation (CV). The ROC curve analysis was performed to determine the optimal cut-off based on the Youden index to establish the best combination of sensitivity and specificity. The agreement between two operators was assessed using the Cohen’s kappa. The analyses were performed with STATA software version 14.0 (College Station, TX: StataCorp LLC) and GraphPad Prism 8.0.2. (GraphPad Software, San Diego, CA, USA).

## 3. Results

Titration of the ND-O-BSA antigen was performed by ELISA, using a positive internal control, a pool of positive sera from LL leprosy patients highly sero-reactive to ND-O-BSA by ELISA. The best performing combination of antigen concentration, serum and secondary antibody dilutions was 25ng of ND-O-BSA per well, 1/300 serum dilution and 1/10,000 secondary antibody dilution (data not shown).

**Figure 1:**
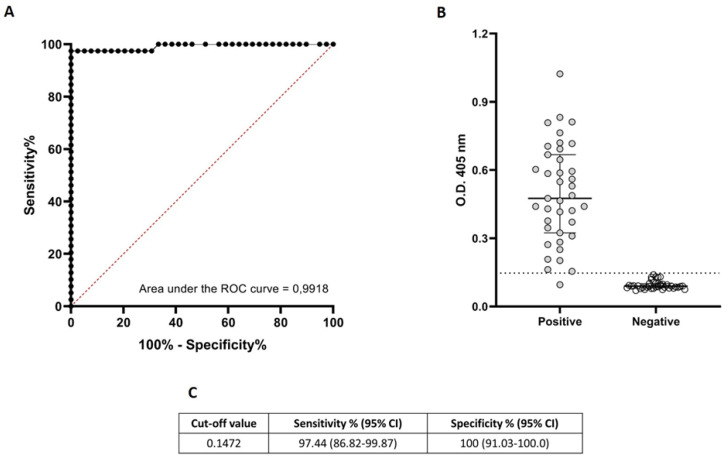
Diagnostic performance of ELISA based on ND-O-BSA.(**A**): Receiver Operating Characteristic (ROC) curves for the detection of antibodies against ND-O-BSA antigen and corresponding areas under the curve (AUC) statistics. The black dotted line shows the mean area under the curve (AUC) plot. The sensitivity and specificity values correspond to the points in the plots. (**B**): Antigen-specific responses of positive (anti-PGL-I seropositive leprosy patients) and negative (healthy volunteers with no suspect of contact with *M. leprae*) control cases. The median with interquartile range is represented by the horizontal bar while the horizontal black dotted line represent the threshold for determining a positive result (OD = 0.147) previously established by the ROC curve. (**C**). threshold (cut-off value) for determining a positive result. CI = 95% confidence interval.

In the first instance we determined the optimal cut-off value (0.147) applying a ROC curve analysis (Figure 1A). The optimal cut-off presented a theoretical sensitivity of 97.4% and a theoretical specificity of 100% (Figure 1B,C).

**Figure 2:**
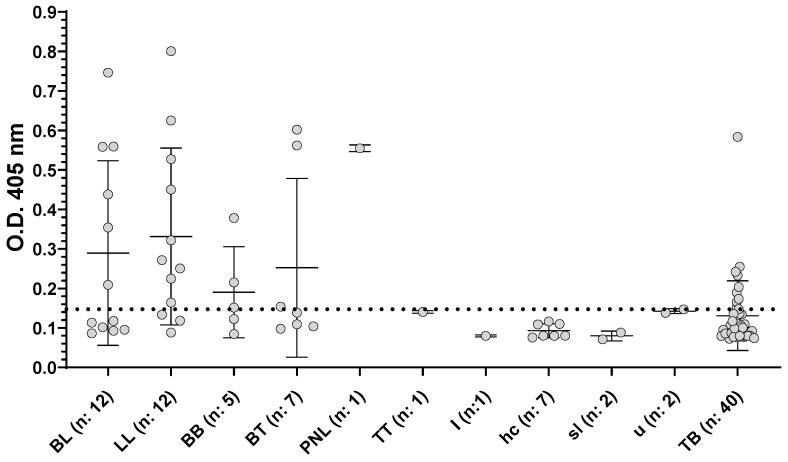
Reactivity of sera towards ND-O-BSA antigens at dilution 1/300. Serum samples from TB patients and SLALT classified following Ridley–Jopling classification, represented as median with interquartile range. BL: borderline lepromatous leprosy; LL: Lepromatous leprosy; BB: mid-borderline leprosy; BT, borderline tuberculoid; PNL, pure neural leprosy; TT, polar tuberculoid; I, indeterminate; hc: household contact; sl: suspected leprosy; u: unknown. Positive/negative cut-off value is shown as a horizontal black dotted line.

We tested the second set of serum samples, and applied the corresponding cut-off value. As shown in Figure 2, the performance of this ELISA varied among the subpopulation of SLALT classified following Ridley–Jopling scale. In this case the statistically determined cut-off, gave a total of 56.4% of positive among patients confirmed diagnosis of leprosy (BL, LL, BB, BT, PNL, TT and I, see Figure 2 and Supplementary 2), of which 50% of the BL patients and 75% of the LL patients gave positive results (Figure 2, Supplementaries 1 and 2). Dividing the patients accordingly to MB and PB we found that, respectively, 54 % and 50% were positive (Supplementary Materials 1 and 2). Applying this cut-off we also observed a 22.5% of positivity among the TB patients.

The variability among the different replicates was low, with CVs% ranging between 0 and 6.99%, (Supplementary 1).

Finally, in order to evaluate the intra-operator variability, the second set of samples was analyzed by two independent operators. Normalizing the results applying the established cut-off previously established, the two operators presented an agreement of 85.25% (Kappa 0.7, *p* ≤ 0.0001). The CVs% among the replicates of the two operators for the second set retained the low variability showed with the first set of sera samples (Supl.1).

## 4. Discussion

ELISA based on PGL-I or ND-O-BSA is widely used in endemic countries, where the reported specificity is 99% [20]. Nevertheless, MB patients are almost universally positive for anti-PGL-I by ELISA whereas positivity for PB patients is low, usually in the range of 20-40%. In fact, in the former form of disease the assay has 87% sensitivity, ranging from 77.1% to 97.3% while it is lower (33%) in the latter, with a range of 15.2–74.4% [24,25,26,27,28,29,30]. Unfortunately, the population heterogeneity described in the literature, in terms of number and kind of participants (i.e., household contacts, infected patients, before or after treatment), makes the diagnostic performance definition difficult. Moreover, important technical information such as the final concentration of the antigen, the time, and the temperature of the incubation steps are not always reported, implying a possible variability in ELISA protocol conditions from different laboratories. All of these factors could significantly influence the test specificity and sensitivity [20]. A study carried out in Spain, another non-endemic European country, reported that the synthetic disaccharide ND-O-BSA is a more sensitive antigen than native PGL-I for monitoring patients during and after treatment [31].

In this study we evaluated the performance of the ELISA based on ND-O-BSA in a non-endemic reality such as Italy. After normalizing the raw O.D., as above described in “Material and Methods”, we found that 46% of sera from the SLALT group gave a positive result. The SLALT group, also including household contact and suspect of leprosy, is more likely to represent the heterogeneous group coming to a non-endemic reality. When we divided the SLALT group into different subgroups we observed that the samples found to be positive were high, up to 75% in LL group. Recently, different studies suggested a possible algorithm to screen the population at risk in endemic areas [32,33,34]. This algorithm is based on the serological screening of the household contacts of leprosy patients followed, in the event of a positive serological test, by a thorough medical examination. If clinical signs suggestive of leprosy are revealed, a PCR test on SSS, a nasal swab and/or a skin or nerve biopsy should always be performed. However, all subjects negative to the first round of serological screening or to the second round (clinical signs and PCR test) will be subjected to one year follow-up [32,33,34]. The application of this algorithm may be very useful in centers of non-endemic countries such as ours, specialized in the management of people coming (migrants) or travelling from endemic countries. The availability of a blood leprosy test to aid in the pool of tests used for the screening of various infectious diseases in migrants from low income countries would simplify the management of these high risk people [35]. In fact, despite the high percentage of the population naturally immune to *M. leprae*, health care professionals in non-endemic country should be constantly aware of leprosy in migrants, travelers and expats that could have come into contact with the pathogen. Indeed, in cases of positivity, the screening will require an overall and precise skin examination to assess loss of sensation in skin lesions by Semmes–Weinstein monofilament tests, palpation of nerves for nerve swelling and pain and muscle weakness or atrophy [36], and eventually other confirmatory laboratory tests for the diagnosis of leprosy. It would be feasible to perform a periodic follow-up and thus, if necessary, promptly start the treatment of those patients that show a potential risk to develop leprosy as well as transmit the pathogen in the community.

In our study, applying the best cut-off, we obtained positive results for 54% of MB and 50% of PB. It is necessary to remember that the SLALT samples used in this study are from patients who were under treatment or have completed at least one cycle of MDT and as a result their titers have likely dropped with time. Others were only suspected of having signs and symptoms of leprosy, or they were a household contact, so these individuals would likely have lower positivity. The fact that the positivity is around 54% shows the ability to use the ND-O-BSA ELISA as a screening tool to identify possible at-risk individuals who will need to be monitored within the next several years. This will allow us to start the treatment promptly and limit the spread of the disease. Moreover, the positive rate among TB patients reported in the literature ranges between 22 and 27.7% [30,37], in agreement with what we observed (i.e., 22.5%).

In order to obtain the optimal performance of the test for a non-endemic center such as ours, the cut-off could be settled accordingly to the real clinical necessity. A different scenario, incrementing the sensitivity or the specificity, could be reached applying different cut-off values.

Due to the limited number of patients coming to our center with a history of being exposed to *M. leprae* or to have been previously diagnosed with leprosy, in this study our SLALT group was limited. Moreover, since the cut-off has to be determined for every single ELISA plate, a known positive and negative control sample needs to be included in every assay. This study should be validated in a larger cohort from a non-endemic country.

## 5. Conclusions

The ELISA based on ND-O-BSA may be considered a good option to use in a non-endemic reality where the at risk population is usually represented by travelers, migrants and expats that already received the diagnosis in the endemic area, and for those who have already received part or full MDT treatment and their contacts. This assay indeed could be a good option to screen the household contacts and to perform a more accurate follow up within the next year, as suggested by the algorithm above specified, or during the treatment follow-up of confirmed leprosy patients.

## Figures and Tables

**Table 1:** Summary of the subjects’ characteristics.

	FIRST SET				SECOND SET			
	Positive		Negative		SLALT		TB	
No. of patients	39		39		50		40	
Origins	Asia	Philippines (39)	Europe	Italy (39)	America	Bolivia (1)	Africa	Benin (1)
						Brazil (11)		Gambia (3)
						Cuba (2)		Ivory Cost (2)
					Africa	Egypt (1)		Morocco (7)
						Ghana (1)		Nigeria (6)
						Guinea (2)		Senegal (1)
						Morocco (1)		Togo (1)
						Nigeria (4)	Asia	India (1)
						Senegal (1)		Sri Lanka (1)
					Asia	Bangladesh (4)	Europe	Italy (8)
						Pakistan (1)		Moldova (1)
						Philippines (4)		Republic of Macedonia (2)
						India (4)		Republic of Serbia (1)
						Sri Lanka (6)		Romania (4)
					Europe	Italy (8)		Unknown (1)
Sex M/F	N/A		16/23		N/A			22/18
Median age in years (IQR)	N/A		37 (30–51.5)		36 (8–73)			35 (22.5–48)
WHO classification	N/A		None		PB (10)			None
					MB (28)			
					Household Contact (7)			
					Suspected (2)			
					Unknown (5)			
Ridley–Jopling classification	BL (11)		None		BL (12)			None
	LL (28)				LL (9)			
					BB (5)			
					BT (7)			
					I (1)			
					LL-R (3)			
					TT- BT (1)			
					PNL (1)			
					Household Contact (7)			
					Suspected leprosy (2)			
					Unknown (2)			
QFT-Plus	N/A		Negative (39)		Neg (3)/ N/A (47)			Neg (2)/Pos (15)/ N/A (23)
*M. tuberculosis* PCR	N/A		N/A		N/A			Positive (40)
Serology results *	Positive (39)		N/A		N/A			N/A

* Positive = positive results also to PGL-I ELISA performed as published in Spencer et al. 2011 [22].

## Data Availability

The datasets generated and analyzed during the current study are available in the present manuscript as Supplemental material.

## Supplementary Materials

The following supporting information can be downloaded at: https://www.mdpi.com/article/10.3390/pathogens11080894/s1, Supplemental 1: Raw data of ELISA-ND-O-BSA; Supplemental 2: Percentage of positive samples.

## References

[1] WHO Leprosy: Situation and Trends. https://apps.who.int/neglected_diseases/ntddata/leprosy/leprosy.html.

[2] WHO (2016). Global Leprosy Strategy 2016–2020.

[3] WHO Leprosy-Key Facts. http://www.who.int/mediacentre/factsheets/fs101/en/.

[4] Massone C., Brunasso A.M.G., Noto S., Campbell T.M., Clapasson A., Nunzi E. (2012). Imported leprosy in Italy. J. Eur. Acad. Dermatol. Venereol..

[5] van Hooij A., Tjon Kon Fat E.M., van den Eeden S.J.F., Wilson L., Batista da Silva M., Salgado C.G., Spencer J.S., Corstjens P.L.A.M., Geluk A. (2017). Field-friendly serological tests for determination of M. leprae-specific antibodies. Sci. Rep..

[6] da Silva M.B., Li W., Bouth R.C., Gobbo A.R., Messias A.C.C., Moraes T.M.P., Jorge E.V.O., Barreto J.G., Filho F.B., Conde G.A.B. (2021). Latent leprosy infection identified by dual RLEP and anti-PGL-I positivity: Implications for new control strategies. PLoS ONE.

[7] WHO (2018). Guidelines for the Diagnosis, Treatment and Prevention of Leprosy.

[8] MSI (Ministero della Salute Italiano) Morbo di Hansen. http://www.salute.gov.it/portale/salute/p1_5.jsp?lingua=italiano&id=203&area=Malattie_infettive.

[9] WHO Diagnosis of Leprosy. https://www.who.int/lep/diagnosis/en/.

[10] Pardillo F.E.F., Fajardo T.T., Abalos R.M., Scollard D., Gelber R.H. (2007). Methods for the Classification of Leprosy for Treatment Purposes. Clin. Infect. Dis..

[11] Ridley D.S., Jopling W.H. (1966). Classification of leprosy according to immunity. A five-group system. Int. J. Lepr. Other Mycobact. Dis..

[12] Nunzi E., Massone C. (2009). La lebbra in Italia. Note di Leprologia.

[13] Gillis T.P. (2015). Mycobacterium Leprae. Molecular Medical Microbiology.

[14] Tatipally S., Srikantam A., Kasetty S. (2018). Polymerase Chain Reaction (PCR) as a Potential Point of Care Laboratory Test for Leprosy Diagnosis—A Systematic Review. Trop. Med. Infect. Dis..

[15] Reibel F., Cambau E., Aubry A. (2015). Update on the epidemiology, diagnosis, and treatment of leprosy. Med. Mal. Infect..

[16] Mohanty P., Naaz F., Katara D., Misba L., Kumar D., Dwivedi D., Tiwari A., Chauhan D., Bansal A., Tripathy S. (2016). Viability of Mycobacterium leprae in the environment and its role in leprosy dissemination. Indian J. Dermatol. Venereol. Leprol..

[17] Spencer J.S., Brennan P.J. (2011). The role of Mycobacterium leprae phenolic glycolipid I (PGL-I) in serodiagnosis and in the pathogenesis of leprosy. Lepr. Rev..

[18] Hungria E.M., Oliveira R.M., Penna G.O., Aderaldo L.C., de Andrade Pontes M.A., Cruz R., de Sá Gonçalves H., Penna M.L.F., Kerr L.R.F.S., de Araújo Stefani M.M. (2016). Can baseline ML Flow test results predict leprosy reactions? An investigation in a cohort of patients enrolled in the uniform multidrug therapy clinical trial for leprosy patients in Brazil. Infect. Dis. Poverty.

[19] Goulart I.M.B., Bernardes Souza D.O., Marques C.R., Pimenta V.L., Gonçalves M.A., Goulart L.R. (2008). Risk and protective factors for leprosy development determined by epidemiological surveillance of household contacts. Clin. Vaccine Immunol..

[20] Espinosa O.A., Benevides Ferreira S.M., Longhi Palacio F.G., Cortela D.D.C.B., Ignotti E. (2018). Accuracy of Enzyme-Linked Immunosorbent Assays (ELISAs) in Detecting Antibodies against Mycobacterium leprae in Leprosy Patients: A Systematic Review and Meta-Analysis. Can. J. Infect. Dis. Med. Microbiol..

[21] De Moura R.S., Calado K.L., Oliveira M.L.W., Bührer-Sékula S. (2008). Leprosy serology using PGL-I: A systematic review. Rev. Soc. Bras. Med. Trop..

[22] Spencer J.S., Kim H.J., Wheat W.H., Chatterjee D., Balagon M.V., Cellona R.V., Tan E.V., Gelber R., Saunderson P., Duthie M.S. (2011). Analysis of antibody responses to Mycobacterium leprae phenolic glycolipid I, lipoarabinomannan, and recombinant proteins to define disease subtype-specific antigenic profiles in leprosy. Clin. Vaccine Immunol..

[23] Savelkoul P.H.M., Catsburg A., Mulder S., Oostendorp L., Schirm J., Wilke H., van der Zanden A.G.M., Noordhoek G.T. (2006). Detection of Mycobacterium tuberculosis complex with Real Time PCR: Comparison of different primer-probe sets based on the IS6110 element. J. Microbiol. Methods.

[24] Chanteau S., Glaziou P., Plichart C., Luquiaud P., Plichart R., Faucher J.F., Cartel J.L. (1993). Low predictive value of PGL-I serology for the early diagnosis of leprosy in family contacts: Results of a 10-year prospective field study in French polynesia. Int. J. Lepr..

[25] Paula Vaz Cardoso L., Dias R.F., Freitas A.A., Hungria E.M., Oliveira R.M., Collovati M., Reed S.G., Duthie M.S., Martins Araújo Stefani M. (2013). Development of a quantitative rapid diagnostic test for multibacillary leprosy using smart phone technology. BMC Infect. Dis..

[26] Lobato J., Costa M.P., Reis E.D.M., Gonçalves M.A., Spencer J.S., Brennan P.J., Goulart L.R., Goulart I.M.B. (2011). Comparison of three immunological tests for leprosy diagnosis and detection of subclinical infection. Lepr. Rev..

[27] Duthie M.S., Raychaudhuri R., Tutterrow Y.L., Misquith A., Bowman J., Casey A., Balagon M.F., Maghanoy A., Beltran-Alzate J.C., Romero-Alzate M. (2014). A rapid ELISA for the diagnosis of MB leprosy based on complementary detection of antibodies against a novel protein-glycolipid conjugate. Diagn. Microbiol. Infect. Dis..

[28] Frade M.A.C., de Paula N.A., Gomes C.M., Vernal S., Bernardes Filho F., Lugão H.B., de Abreu M.M.M., Botini P., Duthie M.S., Spencer J.S. (2017). Unexpectedly high leprosy seroprevalence detected using a random surveillance strategy in midwestern Brazil: A comparison of ELISA and a rapid diagnostic test. PLoS Negl. Trop. Dis..

[29] Leturiondo A.L., Noronha A.B., do Nascimento M.O.O., de Oliveira Ferreira C., da Costa Rodrigues F., Moraes M.O., Talhari C. (2019). Performance of serological tests PGL1 and NDO-LID in the diagnosis of leprosy in a reference Center in Brazil. BMC Infect. Dis..

[30] Jian L., Xiujian S., Yuangang Y., Yan X., Lianchao Y., Duthie M.S., Yan W. (2020). Evaluation of antibody detection against the NDO-BSA, LID-1 and NDO-LID antigens as confirmatory tests to support the diagnosis of leprosy in Yunnan province, southwest China. Trans. R. Soc. Trop. Med. Hyg..

[31] Torres P., Camarena J.J., Gomez J.R., Nogueira J.M., Gimeno V., Navarro J.C., Olmos A. (2003). Comparison of PCR mediated amplification of DNA and the classical methods for detection of Mycobacterium leprae in different types of clinical samples in leprosy patients and contacts. Lepr. Rev..

[32] Barbieri R.R., Manta F.S.N., Moreira S.J.M., Sales A.M., Nery J.A.C., Nascimento L.P.R., Hacker M.A., Pacheco A.G., Machado A.M., Sarno E.M. (2019). Quantitative polymerase chain reaction in paucibacillary leprosy diagnosis: A follow-up study. PLoS Negl. Trop. Dis..

[33] Gama R.S., de Souza M.L.M., Sarno E.N., de Moraes M.O., Gonçalves A., Stefani M.M.A., Garcia R.M.G., de Oliveira Fraga L.A. (2019). A novel integrated molecular and serological analysis method to predict new cases of leprosy amongst household contacts. PLoS Negl. Trop. Dis..

[34] dos Santos D.F., Mendonça M.R., Antunes D.E., Sabino E.F.P., Pereira R.C., Goulart L.R., Goulart I.M.B. (2017). Revisiting primary neural leprosy: Clinical, serological, molecular, and neurophysiological aspects. PLoS Negl. Trop. Dis..

[35] Tosti M.E., Marceca M., Eugeni E., D’Angelo F., Geraci S., Declich S., Della Seta M., Ferrigno L., Marrone R., Pajno C. (2021). Health assessment for migrants and asylum seekers upon arrival and while hosted in reception centres: Italian guidelines. Health Policy.

[36] Frade M.A.C., de Freitas Rosa D.J., Bernardes Filho F., Spencer J.S., Foss N.T. (2021). Semmes-Weinstein monofilament: A tool to quantify skin sensation in macular lesions for leprosy diagnosis. Indian J. Dermatol. Venereol. Leprol..

[37] Gunawan H., Roslina N., Agusni J.H., Kulsum I.D., Makarti K., Hindritiani R., Suwarsa O. (2017). Detection of Anti-Phenolic Glycolipid-I antibody in sera from tuberculosis patients in Bandung, West Java, Indonesia. Int. J. Mycobacteriol..

