# Supplementary material for: ELISA Test Based on the Phenolic Glycolipid-I (PGL-I) of Mycobacterium leprae: A Reality of a Laboratory from a Non-Endemic Country
Source: Pathogens. 2022 Aug 9;11(8):894. doi: 10.3390/pathogens11080894 (PMC9415083; doi:10.3390/pathogens11080894)

Supplemental 2. Percentage of positive samples. Serum samples from MB: Multibacillary; PB= Paucibacillary; LL: Lepromatous leprosy; BL: borderline lepromatous leprosy; Confirmed Leprosy: BL, LL, BB (mid-borderline leprosy), BT (borderline tuberculoid), PNL (pure neural leprosy), TT (polar tuberculoid) and I (indeterminate); SLALT: BL, LL, BB, BT, PNL, TT, I, hc (household contact), sl (suspected leprosy) and u (unknown).

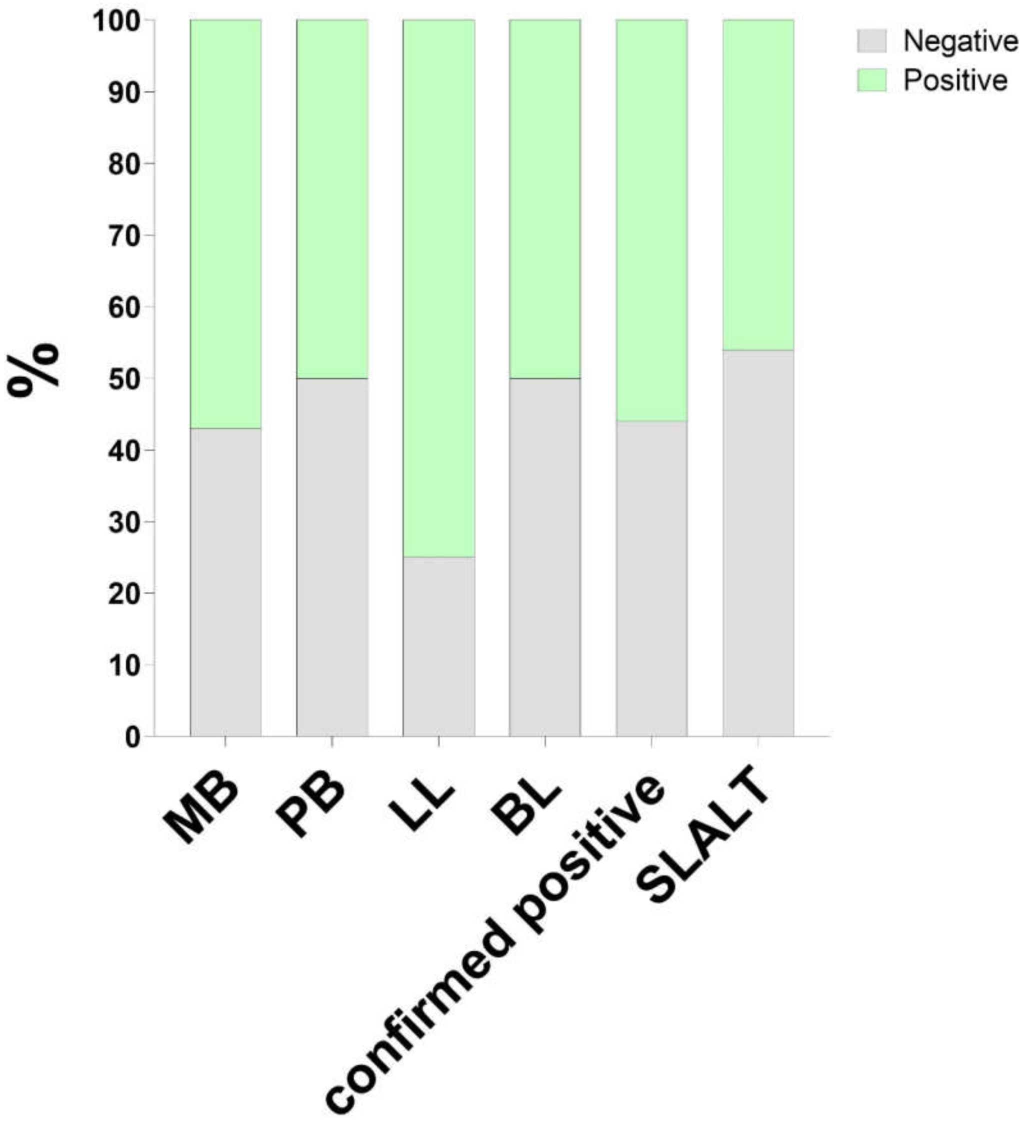

Supplement: Supplementary file 1 [file pathogens-11-00894-s001.zip › Supplemental 2.pdf]
